# Bat species diversity trend along an elevation gradient: A study in Crocker Range Park, Sabah, Borneo

**DOI:** 10.3897/BDJ.9.e72651

**Published:** 2021-11-11

**Authors:** Yen Chi Lok, Vun Gin Siau, Nur Ain Awatif Mohd Kanapiah, Teck Chun Lai, Nur Nisma Haziera Husna Haslan, Nelcilla Nelzah Nukili, Ummu Safiyyah Daud, Amirrah Amat, Juannis Gompoyo, Yit Yu Fred Tuh, Noor Haliza Hasan

**Affiliations:** 1 Institute for Tropical Biology and Conservation, Universiti Malaysia Sabah, Sabah, Malaysia Institute for Tropical Biology and Conservation, Universiti Malaysia Sabah Sabah Malaysia; 2 Department of Zoology, Sabah Parks, Sabah, Malaysia Department of Zoology, Sabah Parks Sabah Malaysia

**Keywords:** Chiroptera, diversity, elevation, Crocker Range Park, Sabah

## Abstract

Bats (Order: Chiroptera) is a recognised group of bioindicators due to their sensitivity towards alterations in their immediate surroundings. With the threats of climate change becoming more severe on a daily basis, it is reasonable to collect data on how bat diversity is influenced by elevation. This will be useful to predict and monitor possible upslope shifting of bat species due to increase in surrounding temperature or anthropogenic pressure. Hence, this study aims to uncover the bat diversity trend at different elevations in Crocker Range Park (CRP), Sabah, Malaysia. Bat trappings were conducted in four substations within this park, covering an elevation spectrum from 450 to 1900 m a.s.l. The overall sampling managed to capture 133 individuals of bats, predominantly Pteropodidae, with the addition of two new species locality records for CRP, *Murinapeninsularis* and *Hypsugovondermanni*. Simple linear regression analyses revealed that both bat diversity and richness have an inverse linear relationship with elevation. Likewise, the Pearson’s correlation value, associating bat diversity with elevation, also shows that they have a negative relationship at r = -0.852. Heterogeneity of habitats explain this trend, as in the lower counterpart of CRP, lowland forests, which are richer in fruit and insect resources persist. Besides, lower land forests have better niche assortment, due to the distinctive layers stratification, allowing bats utilising different guilds to thrive in the same vegetation profile. This study further emphasises the role of CRP to protect most of the bat species found in Borneo, as well as serving as the baseline data for the future studies that look into the impact of temperature increment towards the upslope shifting of the bat population in CRP.

## Introduction

Globally, mountainous forests cover a quarter of the land surface. These uplifted insular areas are also known as ‘Sky Islands’ as they often exist in isolation, with minimal connectivity with one another (Shen 2017). Even so, these are the global hotspots of terrestrial biodiversity, acting as cradles contemporarily and as species’ refuge during climatic fluctuations and uncertainty, specifically during the Quaternary Glacial (Perrigo et al. 2019, Shen 2017, Richter 2008). The complex interaction of altitude with other factors, such as rock features, soil conditions, hydrology and particularly climatic variation, contributes to the segregation into various vegetation zones, consequently creating heterogeneous niches along a gradient slope (Moss and Wilson 1998, Perrigo et al. 2019). Amongst several varieties of mountain forests, tropical mountain forests stand out to be the most speciose (Kessler and Kluge 2008, Richter 2008). This applies, too, to Sabah, Borneo, a tropical Island in Sundaland.

Borneo, in Southeast Asia, is the largest Island in Asia. This Island has been long accredited as one of the less than 40 biological diversity hotspots in the World (Aravanopoulos et al. 2019, Myers et al. 2000). Albeit being slightly less diverse than the Indonesian Island of Sumatra, Borneo still exclusively hosts a larger number of endemic species (Aravanopoulos et al. 2019, Moss and Wilson 1998). Borneo houses the highest mammalian richness in Southeast Asia (de Bruyn et al. 2014), with it being in the top 3% of the most mammal-rich places on Earth (Ceballos and Ehrlich 2006). Contributing to this richness is the availability of diverse geographical features on the Island, as such, mountains (Moss and Wilson 1998).

On Borneo, at least 93 bat species (Mammalia: Chiroptera) have been recorded, accounting for almost 30% of the bat species recorded in the Southeast Asia region (Struebig et al. 2008). This is not surprising, given that the trend of bat richness peaks at the tropic (Willig et al. 2003). Regrettably, it is also projected that Southeast Asia, including Borneo, is vulnerable to losing 40% of its bat species by the year 2100 if the uncontrolled forest destruction persists (Kingston 2008). What deepens the severity of habitat degradation is climate change, where it could result in favourable niches to shift upslope (Shen 2017). For bats, who are effective bioindicators, this shift could be perilous, as they are very sensitive towards changes in environmental factors (Scherrer et al. 2019). In spite of this urgent warning, bats have rarely been a priority when it comes to conservation and lawful protection (Kingston 2013). Anthropogenic threats are not the sole reason why this order of volant mammals should be given scientific attention. Fruit bats are responsible for the pollination and ensuring the dispersal of various fruit trees, including a tenth of Borneo fig species, some of which are keystones (Phillipps and Phillipps 2016). Insectivorous bats, in their own sense, provide insect population control services, concurrently regulating pests that are harmful to crops (Kunz et al. 2011, Mildenstein and Jong 2011, Wilson 2020).

This study focuses on the bats of Crocker Range Park (CRP), a mountainous range in Sabah that covers multiple forest types due to the variation in altitude. Yoh et al. (2020) compared bat ensembles from the different CRP use zones. Several other studies also provided the information on the bats of CRP; however, they are relatively sparse, since they were published in the form of species checklist, based on the trapping done at a singular substation (Salor and Azhar 2018), as preliminary bat surveys (Tuen et al. 2002) or was a minor part of a much larger project (Benda 2010). In an attempt to narrow the gap, this study aims to uncover the trends in bat diversity at different elevations of CRP, based on bat trappings at different substations. This will also be the first study that examines the relationship between bat diversity and richness with altitude using data sourced in this Park. The outcome of this study will be useful in long-term monitoring in the era of rising global temperature, in the sense of detecting niche range-shifting by comparing it with future data.

## Materials and Methods

**Figure 1.**
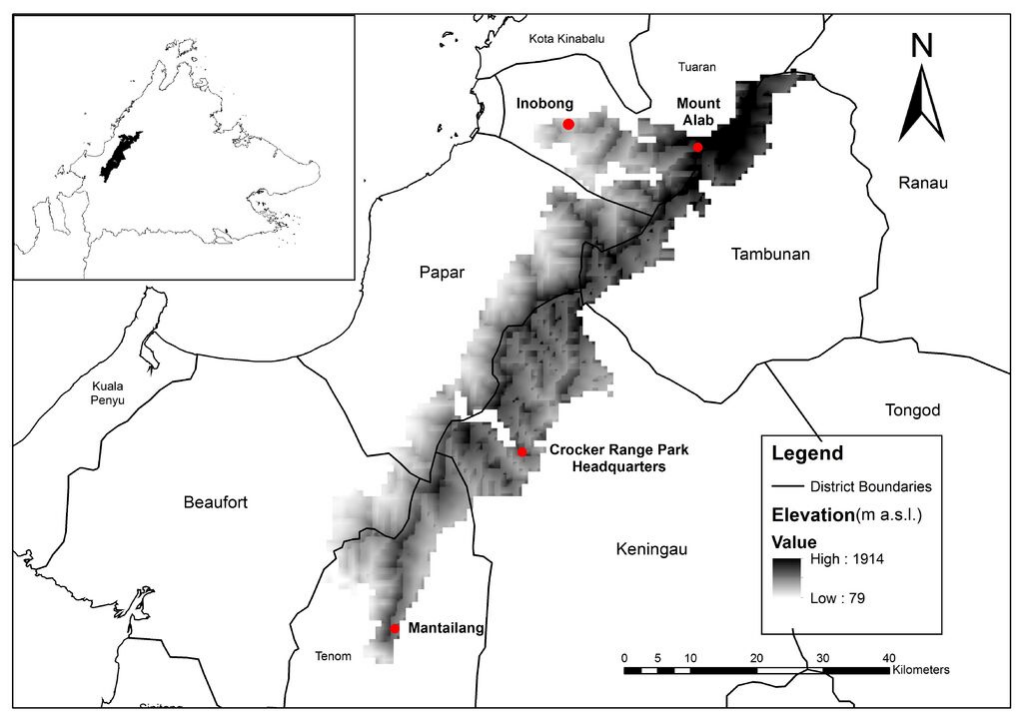
Map of Sabah, Malaysia indicating the location of Crocker Range National Park within the State (top left) and the terrain of the area shaded according to the elevation and the four sampling localities marked with red dots.

**Study Area.** Crocker Range Park (Fig. 1) is the largest gazetted terrestrial national park in Sabah to date. This Park runs parallel to the western coast-line of Sabah, somewhat dividing the State into its eastern and western parts. Size-wise, the Park is approximately 75 km in length and 15 km wide, spanning an area of about 1,399 km^2^. It was first gazetted as a forest reserve in 1969, but was then upgraded to national park status in 1984, due to its importance as a water resource for the west coast of Sabah. Within this Park, there are several types of forest due to the variation in elevation, but predominantly the vegetation profiles found here are hilly dipterocarp forests and montane forests (Yasuma et al. 2003). Bat samplings were accomplished in four localities within the Park area, detailed as follow. For simplicity, forests types of each sampling locality were categorised according to the vegetation profiles used bySaw (2010) andMajit et al. (2014).

**Locality 1: Mantailang Substation.** Mantailang substation is located at the southern part of the Park, within the administrative district of Tenom. The sampling here was conducted between 2 to 13 November 2018 for a duration of two weeks and was part of a Scientific Expedition organised by Sabah Parks. Albeit, only the individuals documented between 2 and 6 November 2018 were included in the statistical analysis of this study. Altitude-wise, Mantailang is about 500 metres above sea level (ma.s.l.), hence the primary vegetation profile here is hill dipterocarp forest.

**Locality 2: Inobong Substation.** The sampling trip to Inobong was 26 to 30 September 2019, where the daily temperature fluctuated between 28°C and 32°C. Inobong substation is part of the District of Penampang and was established in 2003. Situated at the north-western part of the Park, Inobong substation has an altitude level close to Mantailang, at 450 ma.s.l., therefore, both of these substations have the same forest type.

**Locality 3: Crocker Range Park Headquarters.** The main headquarters for Crocker Range Park is located in the District of Keningau, where it has an elevation of 1,000 ma.s.l. The vegetation here is classified as upper hill dipterocarp forest. This was the last sampling site for this study, where data collection happened from 7 to 11 September 2020. During our sampling, the temperature was between 20°C and 25°C.

**Locality 4: Mount Alab Substation.** Mount Alab Substation is the closest substation to Mount Alab, the highest point of the Park. This substation is situated at an elevation level of 1,900 m and higher montane forest occurs here. Within the township of Tambunan, Mount Alab substation is at the north-east of the Park. Sampling at this substation was executed from 10 to 14 August 2020. The temperature ranged from 12°C to 16°C during these dates.

**Bat Sampling.** A total sampling effort of 46 trap stations (eight harp traps and 38 mist nets trapping stations) were deployed to capture bats within the 23 trap nights of this study, which was from 2 November 2018 to 11 September 2021. Generally, bats were captured using mist nets. Ten mist nets (12.5 m x 2.5 m) were erected using extendable poles at each sampling locality. In addition, the sampling effort was supplemented by 2 four-bank harp traps. Traps were set at random points near trails at the substations and were spaced at least 5 to 100 metres away from one another, dependent on the condition and length of the selected trail. In order to maximise trapping possibilities, the choice of trap set-up points was influenced by several factors, namely canopy closure, flight route, availability of nearby fruit trees or other potential habitat (e.g. bamboo trees and rock crevices) and distance from streams and water bodies.

All traps were active for 12 hours each night, from 18.00 h to 06.00 h. To avoid pre-mature death due to strangling and bats escaping by chewing, particularly in mist nets, checking of the traps was done principally once every 30 minutes or every hour, with considerations on trap capture frequency and the length of the trail between 19.00 h and 22.00h and 05.30 h to 06.30 h the next morning, before the traps were closed during the day to avoid trapping aves.

All individuals sampled were identified according to Payne et al. (1985) fundamentally using information on their physical appearance, forearm length (FA) taken using a Mitutoyo digital calliper, along with their ear length (E), tibia length (TB), hind foot length (HF) and tail ventral length (TVL), measured using a Pesola spring balance with weight (W) (Hasan and Abdullah 2011). Identifications were then cross-checked with Phillipps and Phillipps (2016)and the IUCN RedList to ensure that the names are up to date. The sex of the specimen was also recorded and the life stage of the individuals were verified by observing the degree of fusion of the epiphyseal of the joints (Kitchener and Maryanto 1993).

The first three individuals captured for each sampled species were collected and retained as voucher and later deposited at the BORNEENSIS Natural History Collection of the Institute for Tropical Biology and Conservation, Universiti Malaysia Sabah (Appendix). The euthanisation method employed was reviewed and approved by the Animal Ethics Committee of University Malaysia Sabah, under approval no. AEC 004/2020. For samplings prior to 2020, the ethic jurisdiction were part of the consideration in the approval of the research permit from Sabah Wildlife Department. The rest of the individuals, including any pregnant females and those with pups were released at the trapping site, once their basic morphological data had been taken.

**Figure 2.**
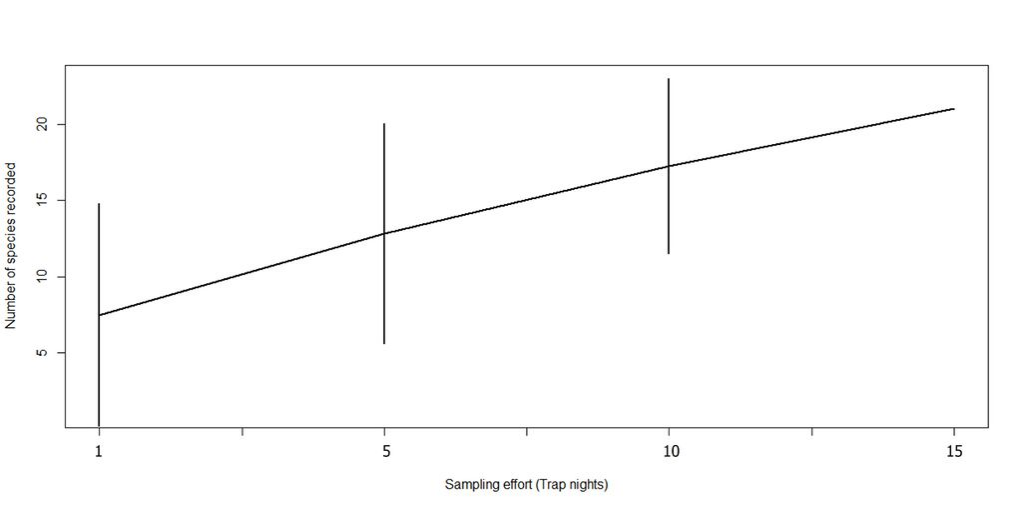
Overall bat species accumulation curve for the bat species captured at Crocker Range National Park.

**Figure 3.**
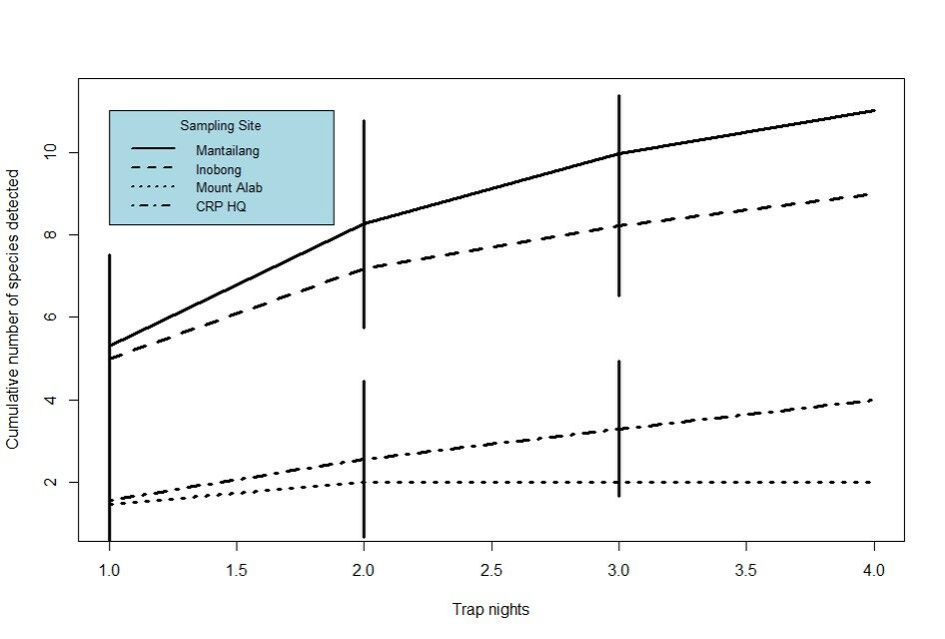
Species accumulation curves for each of the sampling localities.

**Data Analysis.** To obtain a more accurate picture on the sufficiency of the sampling effort in this study, two species accumulation curves were constructed, one for the overall data (Fig. 2), with each sampling site treated as a sampling event. For the purpose of sampling equality, only the first four trap nights at Maintailang substation were included in data analysis. Hereon after, the successive trap nights (7 to 13 November 2020) at this locality was referred as extended sampling for clarity. The second curve (Fig.3) presented the species accumulation for each site, based on trap nights. The sampling completeness was further verified by utilising the values of the species richness estimators, Jack Knife 1 and Chao1 to calculate the percentage of sampling completeness, using the formula as reflected below. The combination of these data is expected to produce a good estimation of the real species richness of the sampling site (Magurran 2004).

Percentage of Sampling Completeness = (Observed species richness / Mean of species estimators) x 100

**Figure 4.**
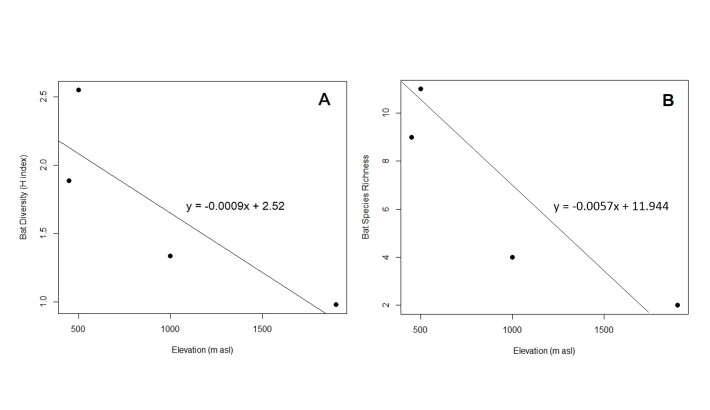
Simple linear regression models of bat diversity across the altitude above sea level of Crocker Range Park. **(A)** Shannon (H) index versus elevation; **(B)** Bat species richness versuselevation.

All data analysis was run in R, version 4.0.2 (R Core Team 2020) using the packages vegan 2.5-6, SPECIES and fossil. The Shannon-Weiner Diversity Index (H) was calculated for each of the respective sites. Subsequently, these values were subjected to the Kruskal Wallis test to identify whether the alpha diversities for the sampling localities differ significantly from one another. To assess the linearity between species richness, the Shannon-Weiner Index with elevation, two simple linear regression models were generated (Figs. 4A and 4B). Next, to verify the magnitude and direction of the linear relationship of bat diversity trend with the change in altitude, Pearson’s Correlation Analysis was conducted on the H index.

## Results

Altogether, 133 individuals of 24 species were detected and identified to species level, based on their morphology.The data from the 16 trap nights included in the statistical analysis (main sampling) are presented in Table 1, whereas for individuals, detected during the extended sampling in Mantailang, are as shown in Table 2. The data from the extended sampling were excluded from analysis for the standardisation of sampling effort across the sampling localities.

Four bat families were accounted, with predominance of Pteropodidae (75 out of 133 individuals [56.4%]), followed by Vespertilionidae (25.6%), Rhinolophidae (16.5%) and Hipposideridae (1.5%). *Cynopterusbrachyotis* was the most frequent species. This species, along with *Cynopterushorsfieldii*, *Kerivoulapapillosa* and *Macroglossusminimus* were the only four species trapped in all sampling localities belonging to different vegetation profiles, specifically upper hill dipterocarp and hill dipterocarp. Several species were also detected as singletons, including *Tylonycterispachypus* and *Murinapeninsularis*, which were trapped within the main sampling. It is noteworthy that the extended sampling yielded three more singletons: *Kerivoulapellucida*, *Hypsugovondermanni* and *Eonycterismajor.*

The cumulative samplings at the four localities did not reach an asymptote (Fig. 2). The percentage of sampling completeness for this study is at 86.9%. Jacknife1 estimated that the samplings should detect 24 bat species, while according to Chao1 analysis, there should be approximately 22 species of bats. None of the individual localities accumulation curves achieves plateau (Fig. 3), with the exception of Mount Alab. Sampling completeness for Mount Alab, Mantailang, Crocker Range Park Headquarters and Inobong stood at 100%, 78%, 73% and 68%, respectively.

In general, sampling localities within the elevation range of hill dipterocarp forest (Mantailang and Inobong) yielded higher bat species richness and diversity than the other forest types (Table 3). In contrast, species richness and diversity were observed to be the lowest at the sampling locality with the highest elevation, which is Mount Alab. Our samplings have also registered two previously undetected bat species in Crocker Range Park, namely *Murinapeninsularis* and *Hypsugovondermanni*, ergo, bringing the total bat species recorded in this National Park to 52 species.

The simple linear regression model Fig. 4(A) indicated that the H index peaked at lower elevation and decreased with the increment of altitudinal gradient. Likewise, bat species richness also exhibit an inverse correlation trend with elevation Fig. 4(B). In spite of the changes in the H index along the gradient, the Kruskal Wallis test analysis revealed that, across the four sampling sites, bat diversity did not differ significantly (p=0.392). Pearson’s Correlation Analysis also indicated that chiropteran diversity correlates negatively with elevation at r=-0.852.

## Discussion

The overall trapping in this study managed to capture 24 species of bats, which is 46.2% of the 52 bat species recorded from Crocker Range Park. Though this is less than half of the bat species recorded from CRP, the sampling has achieved a relatively high completeness at 86.9%. This disparity is explainable by the capture method and the trapping set-up in this study are more sensitive in capturing bats utilising the understorey layer of the forest, but not the other guilds. The traps and nets were all set at ground level, with respective maximum height at 2 m and 10 m, making bats flying above these heights obscure to the traps and nets. To illustrate, *Emballonura* spp. and *Miniopterus* spp. are all distributed within CRP (Yasuma et al. 2003), but were not detected during fieldwork. This is partially because the members of the former genus belong to the open space aerial (canopy) guild, while the latter forages at the edges of tree canopies (Phillipps and Phillipps 2016). Simultaneously, the ratio of harp traps to mist nets (approximately 1:5) deployed in this study is also worth mentioning. More mist nets were set up in this study to compensate for its lower capture efficiency in comparison to harp traps (Francis 1989) and these two devices are co-utilised to make up the species composition bias (Fukui et al. 2001) that each method has in capturing bats.

The dominance of Pteropodid in the dataset can be explained by the presence of banana trees (*Musa* spp.) in three of the sampling localities – Mantailang, Inobong and the headquarters of Crocker Range Park. Musaceae, irrespective of species, provide resources to fruit bats in the form of fruit and flower (Aziz et al. 2021). This family of non-seasonal fruiting plant has several mechanisms facilitating its interaction with bats, such as night-time flowering, emission of bat attracting scent and easy accessibility of parts consumed by bats (Buddenhagen 2008). This explains the abundance and variety of fruit bats captured in the proximity of banana trees. Moreover, several reports (Kumaran et al. 2006, Phillipps and Phillipps 2016) have also suggested that wild bananas are the fall-back materials for bats when food is scarce.

For individual sampling sites, Inobong shows the lowest sampling completeness of all sampling sites, at 67.9%; while Mount Alab has achieved 100% sampling completeness. The difference in these values is due to the standardisation of sampling effort across all sites with the effort of ten mist nets and two harp traps that were actively trapping for four sampling nights. Furthermore, the attainment of complete sampling requires longer sampling periods and coverage for landscapes with more diverse and varied communities of bats (Chao and Jost 2012, Moreno and Halffter 2000). On the contrary, at Mount Alab, where bats are relatively scarce, as indicated by the low trapping rate (Table 1), the sampling completeness curve (Fig. 3) flattened after day 2 of the sampling.

This study contributed to the list of bat species housed by CRP with two new records, namely, *Murinapeninsularis* (Orange tube-nosed bat) and *Hypsugovondermanni* (White-winged Pipistrelle). The latter, a rare species, was trapped during the extended sampling period at Mantailang substation. Within Sabah, *Hypsugovondermanni* has only been reported in Banggi Island previously (Phillipps and Phillipps 2016). Overall, one Bornean endemic (*Aethalopsaequalis*) and a protected species (*Hipposiderosdyacorum*), listed under Schedule 2 of Wildlife Conservation Enactment 1997, were recorded in this study, hence emphasising further the role of Crocker Range Park as a crucial site for Bornean bat species conservation and protection.

Salor and Azhar (2018) did their survey at Ulu Senagang substation that bears a synonymous forest profile with Mantailang and Inobong, as well as sitting on the same altitude level. Since the sampling effort channelled in Salor and Azhar (2018) is the same as the effort for individual sites in this study, it is pertinent to make direct comparison on the species registered by Salor and Azhar (2018) with the species captured in both Mantailang and Inobong. The samplings in Mantailang and Inobong recorded all the species listed by Salor and Azhar (2018), except for one Emballonurid (*Saccolaimussaccolaimus*). Another study by Yoh et al. (2020), also sampled bats in CRP, particularly in Inobong, Mahua and Malungung Control Post, spanning elevations between 500 m to 1 000 m a.s.l. However, detailed comparison stratified according to elevation could not be made, as data presented by Yoh et al. (2020) were segregated by the type of use zones. Yoh et al. (2020), with the supplementary usage of stacked nets, hand netting and acoustic recording, documented 30 species of five chiropteran families, a figure higher than this study. All families recorded by Yoh et al. 2020 were also captured in this study, except for Nycteridae.

The negative correlation and the inverse linear relationship between bat diversity and elevation demonstrated that bat species documented at the lower elevation spectrum of CRP is much more varied and this plummets as it moves to higher grounds. Similar trends in bat richness and diversity have also been observed in several studies at different mountainous systems (Kaňuch and Krištín 2006, Linden et al. 2014, Coelho et al. 2018), in spite of covering narrower altitudinal scopes at 730 m - 1 820 m a.s.l. (Coelho et al. 2018), 956 m -1 745 m a.s.l. (Linden et al. 2014) and 350 m - 1350 m a.s.l. (Kaňuch and Krištín 2006). This finding is also in agreement with the meta-analysis done by McCain (2007) who concluded that, for permanently humid mountains, bat richness and diversity are lower at the higher counterparts of a mountain.

The decline in chiropteran diversity as it shifts towards upper montane forest is explainable by the differences in its vegetation structures. As for Mantailang and Inobong, where hill dipterocarp forest persists, both generally showed similar features as lowland tropical forests. Here, the vertical profile of the forest can be conspicuously divided into three layers, namely the canopy, the understorey and the forest floor, which remains up to 800 m a.s.l. (Saw 2010), in contrast to the forest structure observable at Mount Alab. Due to the overlapping of some features with lowland forest, the hill dipterocarp forest also retains part of the extensive plant diversity of the lowland forest, which include wild fruit species (World Wildlife Fund for Nature 2020). For instance, *Syzygium* spp., *Ficus* spp. and *Musa* spp. that are consumed by frugivorous and nectarivorous bats (Aziz et al. 2021). Entomological populations also flourish well in areas with warmer temperatures due to their ectothermic nature (Syukur et al. 2018). Consequently, all these add up to stable food resources, assortment of niches and foraging guild, thus supporting a diverse range of bats.

In addition to the heterogeneity of forest profiles along the elevation gradient, another reasonable causation to the decreasing trend of bat diversity and richness is the variation of temperature at different elevations in the Park. Michaelsen (2016) and McCain (2007) found that areas with higher temperatures hosts a more assorted group of bats. Furthermore, a publication by Willig et al. 2003, referring to the latitudinal gradient diversity, also backs a similar notion. Chiropterans, in general, show preferences towards higher temperature, as they have a poorer thermoregulatory system than other mammalian groups (Stones and Wiebers 1965). However, this particular variable is not being tested in this study and is only serving as a probable factor in play. It is observed at Mount Alab, where the ambient temperature is of the coldest (12-15°C during our sampling) as opposed to Mantailang and Inobong (28-32°C). Hence, there is a need for further investigation to verify the effect of temperature to bats diversity in the case of CRP.

The bat diversity is moderate at the Headquarters of CRP, Keningau, corresponding to the Shannon Indices of the other substations. It is noted that at mid-elevation of 1 000 m a.s.l., upper hill dipterocarp forest is the transitional zone between the lowland and the highland forest (Majit et al. 2014), simultaneously maintaining some of the fruit bearing trees (Fitri et al. 2017) and fruit bat species observable in the lower counterpart of the Park, such as *Cynopterusbrachyotis* and *Macroglossusminimus*. Here, the majority of the bats trapped were Pteropodidae (12 out of 13 individuals were Pteropodidae), which are most likely to be contributed by the fruiting and maturing of strangler figs in this locality during our sampling period.

Although having the lowest bat species richness and diversity within the surveyed elevational range, it is noteworthy that the two species (*Aethalopsaequalis* and *Rhinolophusluctus*) captured in Mount Alab were not sampled elsewhere in CRP sites explored in this study. *Aethalopsaequalis* is an established montane species, confined exclusively to higher altitude (Phillipps and Phillipps 2016). Unlike the former, *Rhinolophusluctus* is not a montane species, but is rather known to occupy a broad range of elevations with an upper limit at 1 600 m a.s.l. (Thong et al. 2018). With the detection of three individuals at Mount Alab (1900 m a.s.l.), this study is the first to report *R.luctus* at an altitude close to 2 000 m a.s.l. This could mean that the current knowledge on this considerably rare species is still lacking or the probable upslope shifting has already occurred for this particular species. At the moment, there is yet to be any documentation on the range shifting of *R.luctus*. A meta-analysis by Chen et al. (2011)did tabulate the evidence of the shifting of mammalian species to higher elevation (approximately 100 m of range upshifting) due to climate change. This, coupled with the report (Ministry of Natural Resources and Environment Malaysia 2015) that estimated the annual increase of mean temperature in Sabah to be at 0.2°C, the latter scenario is more fitting to explain this discovery.

## Conclusion and recommendations

This study has proven that Crocker Range Park has the capacity to protect a large diversity of bats, by providing heterogeneous forest profiles accounting for different bat species’ guild and specific niche requirements. For future research, it is recommended bat samplings in all the ecosystems available within a mountainous range should give a more comprehensive picture on the diversity trend of the gradient slope. The employment of acoustic techniques would also be useful, principally to sample species that cannot be trapped by the capture methods employed in this study. The data from this study will be useful serving as the baseline data to monitor the shifting of bat habitats due to warming of the climate.

## Appendix

The BORNEENSIS number, localities and some morphological data of the specimens collected during the field samplings of this study are presented (Table 4). The specimens are currently deposited at the wet vertebrate BORNEENSIS collection of Institute for Tropical Biology and Conservation, Universiti Malaysia Sabah.

## Figures and Tables

**Table 1.** Species captured during the samplings of this study and frequency of capture per site and IUCN status categories (LC- Least concern; NT- Near threatened).

**Family/ Species**	**IUCN Status**	**Mantailang**	**Inobong**	**Mount Alab**	**CRP HQ**	**Total individuals captured**
**Rhinolophidae** *Rhinolophusacuminatus* *Rhinolophusborneensis* *Rhinolophusluctus* *Rhinolophussedulus* *Rhinolophustrifoliatus*	LC LC LC NT NT	5 4 0 0 3	0 0 0 4 0	0 0 3 0 0	0 0 0 0 0	5 4 3 4 3
**Pteropodidae** *Aethalopsaequalis* (E) *Balionycterismaculata* *Cynopterusbrachyotis* *Cynopterushorsfieldii* *Cynopterusminutus* *Macroglossusminimus* *Megaeropsecaudatus*	LC LC LC LC LC LC LC	0 0 8 5 2 1 0	0 2 6 0 11 0 3	8 0 0 0 0 0 0	0 0 8 3 0 1 0	8 2 22 8 13 2 3
**Hipposideridae** *Hipposiderosdyacorum*	LC	2	0	0	0	2
**Vespertilionidae** *Kerivoulahardwickii* *Kerivoulaintermedia* *Kerivoulapapillosa* *Glischropustylopus* Murinapeninsularis* Tylonycterisrobustula *Tylonycterispachypus*	LC NT LC LC LC LC LC	1 3 0 0 1 0 0	0 0 1 1 0 15 1	0 0 0 0 0 0 0	0 0 1 0 0 0 0	1 3 2 1 1 15 1
Total individuals captured per site		35	44	11	13	
Trapping rate (No. of individuals per trap night)		8.75	11.00	2.75	3.25	

CRP HQ is an abbreviation for Crocker Range Park Headquarters. Asterisk (*) indicates new species record in Crocker Range Park. 'E' is an indication for species endemic to Borneo Island. The data presented for Mantailang is only for the first four nights of the sampling.

**Table 2.** Bats species captured during the extended sampling in Mantailang and IUCN status categories (LC- Least concern; NT- Near threatened; DD- Data deficient).

**Family**	**Species**	**IUCN Status**	**Number of individuals detected**
Rhinolophidae Pteropodidae Vespertilionidae	*Rhinolophustrifoliatus* *Rhinolophusborneensis* *Macroglossusminimus* *Cynopterusbrachyotis* *Cynopterusminutus* Eonycterismajor** (X) Pipistrellusstenopterus** (X) *Kerivoulahardwickii* *Kerivoulaintermedia* Kerivoulapellucida** (X) Hypsugovondermanni** (X) *Glischropustylophus*	NT LC LC LC LC DD LC LC NT NT DD LC	1 2 7 5 4 1 2 3 1 1 1 2

Asterisks (*) indicate new species record in Crocker Range Park and IUCN status categories: LC- Least concern; NT- Near threatened; DD- Data deficient. 'X' are species which were not detected in the main samplings.

**Table 3.** Elevation, trap nights, species richness and H index for each sampling locality

**Site**	**Inobong**	**Mantailang**	**CRP HQ**	**Mount Alab**
Elevation (m a.s.l.)	450	500	1 000	1 900
Forest type	Hill dipterocarp	Hill dipterocarp	Higher montane	Upper montane
Trap Nights	4	4	4	4
Species Richness	9	11	4	2
Shannon- Weiner Index (H)	1.886	2.564	1.330	0.980

**Table 4.** Specimens taken and deposited into the BORNEENSIS collection

**BORNEENSIS No.**
	**Locality**	**Family**	**Species**	**Sex**	**FA**	**Tibia**	**Hind foot**	**Weight**
MAL10492	Mantailang	Pteropodidae	*Cynopterusbrachyotis*	F	66.97	27.13	10.63	41.00
MAL10482	Mantailang	Pteropodidae	*Cynopterushorsfieldii*	F	65.35	25.68	10.17	36.00
MAL10467	Mantailang	Pteropodidae	*Cynopterushorsfieldii*	M	73.65	28.84	6.98	60.00
MAL10488	Mantailang	Pteropodidae	*Cynopterushorsfieldii*	F	73.22	27.73	12.31	51.00
MAL10489	Mantailang	Pteropodidae	*Cynopterusminutus*	F	58.90	22.30	9.80	25.00
MAL10491	Mantailang	Pteropodidae	*Cynopterusminutus*	M	58.21	17.07	8.63	24.00
MAL10498	Mantailang	Pteropodidae	*Cynopterusminutus*	F	56.04			
MAL10499	Mantailang	Vespertilionidae	*Glischropustylopus*	F	29.24	12.87	6.87	3.60
MAL10486	Mantailang	Hipposideridae	*Hipposiderosdyacorum*	M	41.13	16.58	6.92	6.00
MAL10487	Mantailang	Hipposideridae	*Hipposiderosdyacorum*	F	41.89	17.48	7.70	7.00
MAL10478	Mantailang	Vespertilionidae	*Kerivoulaintermedia*	F	30.47	15.72	5.15	3.50
MAL10481	Mantailang	Vespertilionidae	*Kerivoulaintermedia*	F	31.51	16.14	5.44	4.00
MAL10494	Mantailang	Vespertilionidae	*Kerivoulaintermedia*	M	29.60	15.82	5.23	3.30
MAL10490	Mantailang	Pteropodidae	*Macroglossusminimus*	M	39.74	15.84	9.44	12.00
MAL10501	Mantailang	Pteropodidae	*Macroglossusminimus*	M	38.92			16.20
MAL10500	Mantailang	Pteropodidae	*Macroglossusminimus*	M	42.22			18.20
MAL10480	Mantailang	Vespertilionidae	*Murinapeninsularis*	M	37.54	20.30		9.00
MAL10468	Mantailang	Rhinolophidae	*Rhinolophusacuminatus*	M	50.75	22.72	9.85	15.20
MAL10483	Mantailang	Rhinolophidae	*Rhinolophusacuminatus*	F	50.40	22.00	8.02	14.00
MAL10484	Mantailang	Rhinolophidae	*Rhinolophusacuminatus*	F	50.98	22.35	9.31	13.80
MAL10479	Mantailang	Rhinolophidae	*Rhinolophusborneensis*	M	44.43	18.77	6.96	8.10
MAL10493	Mantailang	Rhinolophidae	*Rhinolophusborneensis*	F	42.35	18.10	6.55	8.00
MAL10495	Mantailang	Rhinolophidae	*Rhinolophusborneensis*	F	43.47	18.98	6.86	8.90
MAL10485	Mantailang	Rhinolophidae	*Rhinolophustrifoliatus*	M	54.67	28.79	10.97	14.50
MAL10496	Mantailang	Rhinolophidae	*Rhinolophustrifoliatus*	F	51.06	25.04	9.79	18.50
MAL10497	Mantailang	Rhinolophidae	*Rhinolophustrifoliatus*	F	52.87	25.36	9.47	13.50
MAL10079	Mount Alab	Rhinolophidae	*Rhinolophusluctus*	M	65.76	29.89	14.90	34.00
MAL10078	Mount Alab	Pteropodidae	*Aethalopsaequalis*	F	45.70	17.05	9.60	18.00
MAL10077	Mount Alab	Pteropodidae	*Aethalopsaequalis*	M	42.89	15.25	8.95	15.50
MAL10080	Mount Alab	Pteropodidae	*Aethalopsaequalis*	M	46.50	16.47	6.81	18.50
MAL10081	Mount Alab	Rhinolophidae	*Rhinolophusluctus*	F	65.76	36.59	13.14	34.50
MAL10082	Mount Alab	Rhinolophidae	*Rhinolophusluctus*	F	68.23	34.26	15.22	38.00
MAL10083	CRP HQ	Pteropodidae	*Cynopterusbrachyotis*	M	62.88	22.34	11.87	35.00
MAL10086	CRP HQ	Pteropodidae	*Cynopterusbrachyotis*	M	57.30	20.41	10.03	30.00
MAL10087	CRP HQ	Pteropodidae	*Cynopterushorsfieldii*	M	66.50	23.84	9.01	43.00
MAL10088	CRP HQ	Pteropodidae	*Cynopterushorsfieldii*	M	68.32	23.46	8.85	39.00
MAL10089	CRP HQ	Vespertilionidae	*Kerivoulapapillosa*	F	47.57	23.65	6.37	10.00
MAL10068	Inobong	Pteropodidae	*Balionycterismaculata*	F	42.63	11.71	6.06	18.00
MAL10058	Inobong	Pteropodidae	*Cynopterusbrachyotis*	F	59.18	14.81	6.61	32.00
MAL10067	Inobong	Pteropodidae	*Cynopterusminutus*	F	57.85	17.51	5.53	26.00
MAL10074	Inobong	Pteropodidae	*Cynopterusminutus*	M	57.12	19.72	4.31	25.00
MAL10069	Inobong	Vespertilionidae	*Glischropustylopus*	M	30.40	12.80	4.07	4.40
MAL10055	Inobong	Vespertilionidae	*Kerivoulapapillosa*	M	45.06	22.34	8.34	11.50
MAL10066	Inobong	Pteropodidae	*Megaeropsecaudatus*	M	54.30	26.80	8.77	19.00
MAL10059	Inobong	Pteropodidae	*Megaeropsecaudatus*	F	53.05	19.69	10.47	23.00
MAL10072	Inobong	Pteropodidae	*Megaeropsecaudatus*	F	50.65	21.24	2.81	17.50
MAL10057	Inobong	Rhinolophidae	*Rhinolophussedulus*	M	46.24	23.05	5.28	12.00
MAL10070	Inobong	Rhinolophidae	*Rhinolophussedulus*	M	44.65	24.43	5.77	9.00
MAL10075	Inobong	Rhinolophidae	*Rhinolophussedulus*	M	44.13	19.60	2.37	9.00
MAL10056	Inobong	Vespertilionidae	*Tylonycterisrobustula*	F	28.13	13.00	8.47	6.10
MAL10071	Inobong	Vespertilionidae	*Tylonycterispachypus*	F	28.52	12.64	3.66	9.00
MAL10073	Inobong	Vespertilionidae	*Tylonycterisrobustula*	F	28.06	11.08	3.56	7.50
MAL10076	Inobong	Vespertilionidae	*Tylonycterisrobustula*	F	27.72	11.57	3.85	7.00
